# Six-year epidemiological analysis of post traumatic endophthalmitis in a Brazilian hospital

**DOI:** 10.1186/s40942-019-0193-8

**Published:** 2019-11-26

**Authors:** Luis Filipe Nakayama, Vinicius Campos Bergamo, Nilva Simeren Bueno de Moraes

**Affiliations:** Department of Ophthalmology, Universidade Federal de Sãso Paulo - EPM, Botucatu St, 816. Vila Clementino, São Paulo, SP 04039-032 Brazil

**Keywords:** Endophthalmitis, Retina, Epidemiology

## Abstract

**Background:**

To evaluate the epidemiology of endophthalmitis cases related to ocular trauma, including visual acuity during and 1 year after trauma, source of trauma and method of treatment.

**Methods:**

A retrospective study analyzed the epidemiological data of patients with a clinical presentation of endophthalmitis after ocular penetrating trauma between January 2012 and January 2017 at Escola Paulista de Medicina/UNIFESP, a hospital in São Paulo, SP, Brazil.

**Results:**

A total of 453 patients with antecedent open globe trauma were evaluated, among these, 30 patients with suspected endophthalmitis. All patients were male. The time interval between trauma and ophthalmological evaluation and collection of vitreous and aqueous material was 1 day in 36.66%, 2–7 days in 43.44%, 7–14 days in 10% and more than 15 days in 10% of patients; 66.66% had positive cultures. 11 patients had intraocular foreign body. One year after trauma, visual acuity was classified as no light perception (NLP) in 33.33%, light perception in 6.66%, hand motion in 13.33%, counting fingers in 13.33%, and better than 20/400 in 20% of patients. Considering presence of intraocular foreign body, initial visual acuity and symptoms onset time, only initial visual acuity showed as better prognostic factor in final visual acuity.

**Conclusion:**

Endophthalmitis is a severe ocular inflammatory condition that may lead to irreversible vision loss. Initially only one patient had visual acuity of NLP, but after 1 year, 33% showed visual acuity of NLP, and only 20% had visual acuity better than 20/400, what is consistent with a severe infection with a guarded prognosis. The high incidence of endophthalmitis after ocular penetrant trauma justifies distinct treatment and greater attention.

## Background

Endophthalmitis is a severe inflammatory ocular disease with profound vision loss that may become irreversible and requires immediate treatment [1, 2]. Endophthalmitis is classified as acute when symptoms occur within 6 weeks after a procedure or late when symptoms occur more than 6 weeks after a procedure [1, 3]. According to etiology, endophthalmitis is further classified as endogenous when the infectious agent originates from the patient via hematological spreading [4] or exogenous when the etiological agent originates externally from the eye by direct inoculation.

Endophthalmitis presents clinically as a decrease in visual acuity, conjunctival injection and hyperemia, ocular pain, eyelid edema, and intraocular inflammatory signs, such as anterior chamber reaction, hypopyon, fibrin and vitreitis [2, 5].

The incidence rate of post-traumatic endophthalmitis can reach 16.5% [6, 7], and the causative agent is normally related to the periocular cutaneous microbiota, but in post-penetrating trauma endophthalmitis, atypical agents have been reported, such as *Bacillus cereus* [2].

Our study objective was to evaluate the epidemiology of endophthalmitis cases related to penetrating ocular trauma, visual acuity during and 1 year after endophthalmitis, the source of ocular trauma, and method of treatment.

## Materials and methods

This retrospective study analyzed epidemiological data of patients undergoing evaluation in the emergency department who were submitted to intravitreous or anterior chamber material collection due to suspected acute endophthalmitis and related antecedents of ocular penetrating trauma from January 2012 to January 2017 at Escola Paulista de Medicina/UNIFESP, a reference hospital in São Paulo, SP, Brazil.

Our study followed the Declaration of Helsinki and was approved by the Escola Paulista de Medicina - São Paulo Federal University Ethics Committee (0060/2018).

Data from vitreous and anterior chamber analysis were obtained from the records of the Ophthalmology and Microbiology Laboratory. Epidemiological and ophthalmological data were collected from medical records.

For penetrating globe trauma, our hospital followed a protocol of immediate hospital admission and subsequent administration of systemic prophylactic intravenous antibiotics as Cefalotin 1 g twice/day and Gentamicin 80 mg three times/day until surgery. Analgesics and nausea relief drugs were prescribed as needed. Patients who are allergic to this treatment or had renal insufficiency received alternative antibiotics or adequate doses.

We performed a complete ophthalmological exam of the contralateral eye; no topical eye drops were used in the injured eye. All patients underwent orbit and cranial computed tomography with thin slices to evaluate the possibility of other lesions and fractures and to evaluate any intraocular foreign body.

In penetrant ocular trauma surgical repair was performed as soon as possible, according to the patient's systemic clinical condition. Corneal suture was done with nylon 10.0 and scleral sutures with *Vicryl* 7.0 with removal of the lens when occurs capsule rupture and removal of the foreign body when possible. Systemic antibiotics were maintained throughout hospitalization, and topical moxifloxacin 0.5% and 1% prednisolone acetate were prescribed postoperatively. Systemic antibiotics were not routinely prescribed after hospital discharge.

When patients presented clinical symptoms and signals of endophthalmitis, 0.2 mL of vitreous and aqueous material were collected and empirical intravitreal antibiotics were injected in order to treat gram-positive and gram-negative bacteria [5]. In our hospital, the standard intravitreal injection is 0.05 mL of Vancomycin 1 mg and 0.05 mL of Ceftazidime 2.25 mg.

In patients with post-trauma endophthalmitis, we compared age, gender, eye, time until first evaluation, culture results, etiological agent, trauma mechanism, and visual acuity during endophthalmitis and 1 year after endophthalmitis. We evaluated pre-operative visual acuity and trauma agents as prognostic factors.

## Results

During the 6-year period, 453 patients with open globe trauma with or without an intraocular foreign body were evaluated in the Escola Paulista de Medicina/UNIFESP emergency department. Of these 453 patients, 30 (6.62%) presented a clinical exam compatible with acute infectious endophthalmitis. The mean age of the patients was 42.26 years, with a standard deviation of 15.61, a minimum of 11 years, and a maximum of 80 years. All patients were male, with involvement of the right eye in 11 patients (36.66%) and the left eye in 19 patients (63.33%). The time between trauma and ophthalmological evaluation and collection of vitreous and aqueous material was 1 day in 36.66% of patients, 2 to 7 days in 43.44% of patients, 7 to 14 days in 10% of patients, and more than 15 days in 10% of patients (Table 1).

**Table 1:** Epidemiological and trauma parameters

Variable	No. of patients (%)
Eye	
Right	11 (36.66%)
Left	19 (63.33%)
Sex	
Male	30 (100%)
Female	0 (0%)
Age	
18 or younger	2 (6.7%)
Older than 18	28 (93.3%)
Mean	42.26 years
Composition of traumatic agent	
Metal	22 (73.3%)
Wood	3 (10%)
Plastic	1 (3.3%)
Concrete	1 (3.3%)
Glass	1 (3.3%)
Non-identified	2 (6.7%)
Time from injury to material collect	
Less than 1 day	11 (36.7%)
Between 2 and 7 days	13 (43.3%)
Between 7 and 14 days	3 (10%)
After 15 days	3 (10%)
BCVA of NLP	
Initial	1 (3.3%)
After 1 year	10 (33.3%)

Of the 30 patients with suspected acute infectious endophthalmitis, 20 (66.66%) presented positive cultures. Three of these 20 cultures were positive only in aqueous culture (15%), 15 only in vitreous culture (75%) and 2 in both aqueous and vitreous culture (10%). The responsible etiological agent was coagulase-negative staphylococci in 12 cultures (40%) and *Bacillus cereus* in 3 cultures (10%). Other cultures were positive for *Lactococcus lactis* (3.33%), *Pseudomonas fluorescens* (3.33%), *Serratia marcescens* (3.33%), *Enterococcus faecalis* (3.33%), *Enterococcus faecium* (3.33%), *Aerococcus viridans* (3.33%) and *Sphingomonas paucimobilis* (3.33%). Five patients (16.66%) presented more than one positive agent in culture.

The traumatic agent responsible for penetrant ocular trauma was metal in 22 patients (73.33%), wood in 3 patients (10%), glass in 1 patient, plastic in 1 patient, and concrete in 1 patient. In 2 patients, the responsible material could not be identified.

Eleven patients (36.66%) presented intraocular foreign body (IOFB) after ocular trauma. We do not usually use intravitreous antibiotics previous to endophthalmitis suspect, neither when there are IOFB.

Eleven patients (36.66%) needed vitrectomy. From 30 patients, 29 primarily had sutured cornea and 1 had self-sealant lesion.

Initial visual acuity (VA) during endophthalmitis was between 20/20 and 20/400 in 5 patients (16.66%), counting fingers in 4 patients (13.33%), hand motion in 8 patients (26.66%), light perception in 7 patients (23.33%) and no light perception (NLP) in 1 patient. Four patients had incomplete medical records with undefined initial visual acuity. One year after endophthalmitis, visual acuity was NLP in 10 patients (33.33%), light perception in 2 patients (6.66%), hand motion in 4 patients (13.33%), counting fingers in 2 patients (13.33%) and better than 20/400 in 6 patients (20%).

When considering only patients with IOFB, during endophthalmitis episode, 1 patient had initial NLP initial visual acuity and, 1 year after, 5 patients had NLP final VA (45%).

We applied liner regression methodology for risk factor analysis and considered significance of 5%. To apply linear regression we used R software version 3.6.0 and applied EVS visual acuity score.

Analyzing presence of intraocular foreign body, initial visual acuity and symptoms onset time, only initial visual acuity showed as better prognostic factor (p 0.002) in final visual acuity.

## Discussion

Endophthalmitis is a severe ocular inflammatory condition associated with irreversible vision loss and high morbidity. Epidemiological analysis are fundamental to better understand the associations among the trauma mechanism, trauma agent, time until treatment and visual acuity in order to establish a visual prognosis, prevent future trauma, and appropriately treat similar cases.

This is the first Brazilian study to evaluate cases of endophthalmitis after penetrating ocular trauma in a hospital. Variable prevalence rates of 1–17% for acute endophthalmitis after ocular trauma with an intraocular foreign body have been reported in the literature [8], similar to our prevalence rate of post-penetrating trauma endophthalmitis of 6.62% at Escola Paulista de Medicina over a period of 6 years [9].

All patients with post-penetrating trauma acute endophthalmitis were male in our study, corroborating previous reports of higher prevalence in male patients [10].

In all cases, endophthalmitis symptoms started acutely, within 15 days after the ocular trauma episode and within 7 days in the majority of patients (80%), which reflects the higher virulence and severity of infectious endophthalmitis after penetrating ocular trauma.

Nearly 70% of our patients presented positive cultures, compatible with the results of the Endophthalmitis Vitrectomy Study (EVS), although EVS evaluated endophthalmitis occurring after cataract surgery, which is a distinct disease compared to trauma-related endophthalmitis [3]. The sensitivity of vitreous culture was higher than that of aqueous culture (56.6% vs. 6.6%), in contrast to the results of a study by Baza [11], probably due to differences in the characteristics of trauma-related endophthalmitis and the collected materials. The culture results indicated more than one pathogen in 16% of our patients, in contrast to acute endophthalmitis after cataract surgery [3] and post-intravitreal anti-VEGF injection [2], which are usually characterized by a single agent. These results confirm that penetrating traumas are associated with more virulent and severe infections.

Although initially only one patient exhibited visual acuity of NLP, after 1 year, 33% showed visual acuity of NLP, and only 20% had visual acuity better than 20/400, further indicating severe infection with a reserved prognosis. When considering patients with IOFB, rates of NLP 1 year after endophthalmitis were even higher (45%). These rates of traumatic and post-traumatic endophthalmitis and infection are probably not related to the tropical environment but to the characteristics of the trauma and the agents related to ocular trauma.

We are the first to report the poor prognosis of visual acuity in post-penetrating trauma-related endophthalmitis, with an evaluation of the trauma agents and the results of microbiological analysis. Our results confirm that acute infectious endophthalmitis has a reserved prognosis and its prognosis is even poorer after penetrating ocular trauma. The high incidence of endophthalmitis after penetrating ocular trauma justifies distinct treatment and greater attention in these cases.

We also report initial visual acuity during endophthalmitis episode as a prognostic factor in final visual acuity.

This study has limitations, such as its retrospective nature and the inclusion of a single hospital's experience. The lack of access to prompt ophthalmological emergency care in Brazil contributes to the time lapse between trauma and evaluation. More studies are needed to evaluate antibiotic efficacy and improve antibiotic delivery to reduce infectious endophthalmitis after ocular penetrating trauma.

## Conclusion

Endophthalmitis is a severe ocular inflammatory condition that may lead to irreversible vision loss. The high incidence of endophthalmitis after ocular penetrant trauma justifies distinct treatment and greater attention in these cases.

## Data Availability

The datasets generated during the current study that are used to calculate the primary outcome are available upon reasonable request from the corresponding author.

## Abbreviations

NLP: no light perception
EVS: Endophthalmitis Vitrectomy Study
VEGF: Vascular Endothelial Growth Factor
IOFB: intraocular foreign body
VA: visual acuity
